# A slowly-growing solitary skip lymph nodule of occult gastric cancer above the neck of pancreas: a case report

**DOI:** 10.1186/s40792-020-00895-w

**Published:** 2020-06-15

**Authors:** Yuhao Tang, Xiaowei Sun, Li Xu

**Affiliations:** 1 Department of Liver Surgery, Sun Yat-sen University Cancer Center, 651 Dongfeng East Road, Guangzhou, 510060 People’s Republic of China; 2 State Key Laboratory of Oncology in South China, Guangzhou, People’s Republic of China; 3 Collaborative Innovation Center for Cancer Medicine, Guangzhou, People’s Republic of China; 4 Department of Gastric Surgery, Sun Yat-sen University Cancer Center, Guangzhou, People’s Republic of China

**Keywords:** Occult gastric cancer, Lymph node metastasis, Skip lymphatic metastasis

## Abstract

**Background:**

Skip lymphatic metastasis (SK) is an exceptional and characteristic pattern of lymph node metastasis in gastric cancer (GC) with infrequent incidence. This is an extremely rare report of occult gastric cancer with solitary skip lymphatic metastasis as the initial and primary observation.

**Case presentation:**

A 61-year-old woman, who complained of epigastric discomfort for several years, presented a solitary nodule upon pancreas neck examination by CT without performance on the primary lesion, even gastroscopy. During the preoperative 4-month follow-up, the nodule stayed stable without any therapy. The postoperative pathological examination confirmed the consistent diagnosis of gastric adenocarcinoma between the nodule and the stomach lesion, which was found by preoperative random biopsy of the mucosa.

**Conclusions:**

This case highlights the concentration on vigilance to the SK of GC and a closer observation for intra-abdominal nodules, even radiological suspicion of a benign lesion.

## Background

Gastric cancer (GC) is the fifth most frequently diagnosed cancer and the third leading cause of cancer-related death [1]. Due to the vague clinical manifestations and signs, GC patients are usually diagnosed at advanced stages. The lymphatic system is one of the main routes for GC, and lymph node metastasis (LNM) is defined as a vital prognostic factor. In this context, the pretreatment diagnosis of LNM would be helpful for more survival benefits [2]. However, sometimes bypass or skip tiers of LNM can be detected far from the original tumor without being detected in the peritumoral area, which is called skip metastasis [3]. Therefore, the existence of skip LNM can frequently cause mistakes or overlooking of the diagnosis of GC, especially of clinical occult GC, which is characteristic of the metastasis as the first symptom and the overlooking of the primary lesion.

We now report our experience with a case of a slow-growing solitary metastatic lymph node above the neck of the pancreas without performance on and observation of the primary GC lesion.

## Case presentation

**Fig. 1:**
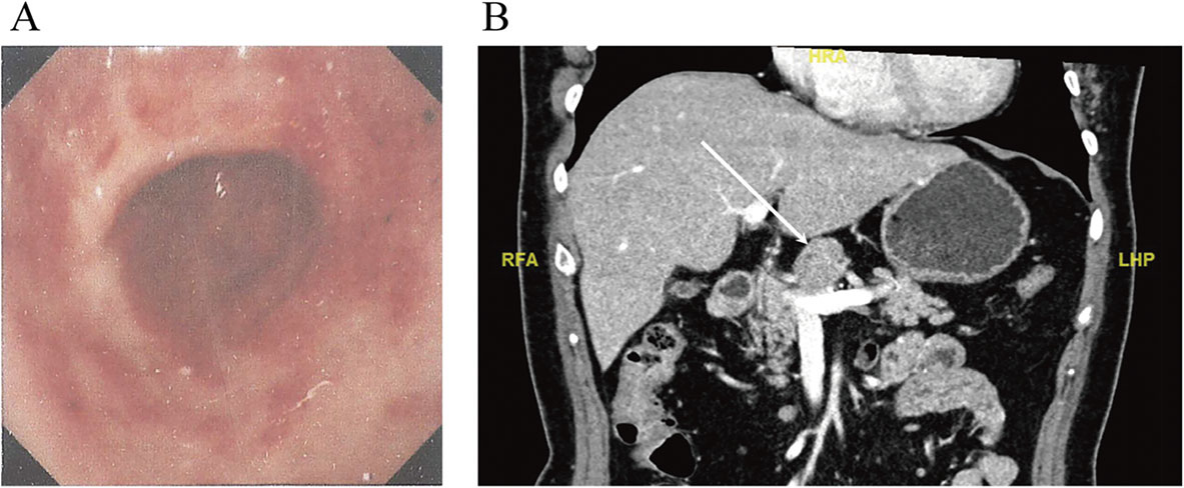
The first preoperative tests. **a** The first gastroscopy that was taken at another hospital (scanned version of printed report). Slight superficial mucosal erosion of the gastric antrum. **b** The contrast-enhanced abdominal CT. A solitary nodule above the neck of the pancreas, size 29 mm × 27 mm, labeled by the white arrow

**Fig. 2:**
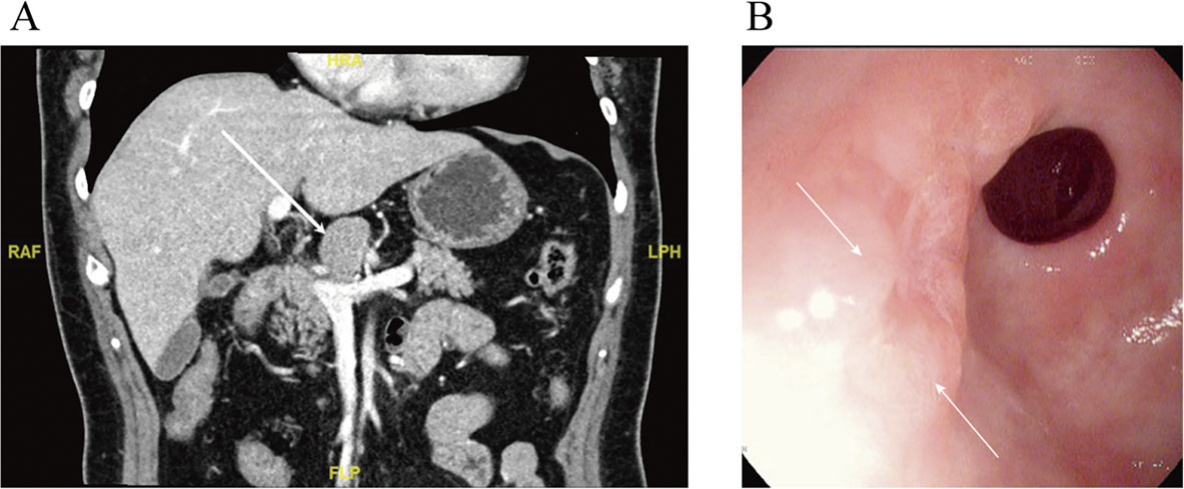
The last preoperative tests. **a** The latest contrast-enhanced abdominal CT. Stable solitary nodule (32 mm × 25 mm), labeled by the white arrow, without other abnormal findings. **b** The retaken gastroscopy found two bumps with mucosa erosion near the pylorus on the anterior gastric antrum, and a biopsy was taken around this area

A 61-year-old woman complained of epigastric discomfort for several years. She received gastroscopy that indicated a slight superficial mucosal erosion of the gastric antrum with negative *Helicobacter pylori* (Fig. 1a), and ultrasonography found an upper abdominal mass. Contrast-enhanced abdominal computed tomography (CECT) revealed the existence of a solitary nodule above the neck of the pancreas—size 29 mm × 27 mm, clear boundary, uniform density, lobulated, and slowly reinforced with contrast (Fig. 1b). The radiological findings diagnosed the lesion as benign. The patient’s discomfort improved when she took omeprazole for 2 weeks and chose to wait and see instead of undergoing biopsy or operation. A repeat CECT scan was done 3 months later, which showed a slight increase in the size of the solitary nodule (32 mm × 25 mm), with no other abnormal findings (Fig. 2a). The patient had no fever and no loss of weight. Body examination did not reveal any abnormal signs, and no enlarged superficial lymph node was detected. Laboratory analysis showed a slightly increased blood glucose and triglyceride. Tumor biomarkers (carcinoembryonic antigen (CEA), carbohydrate antigen (CA) 19-9, CA 125, and CA 242) and immunological tests (β2 microglobulin, immunoglobulin G4, interleukin-2, interleukin-4, interleukin-6, interleukin-10, interferon-γ, and lymphocyte subsets) were all within the normal range.

**Fig. 3:**
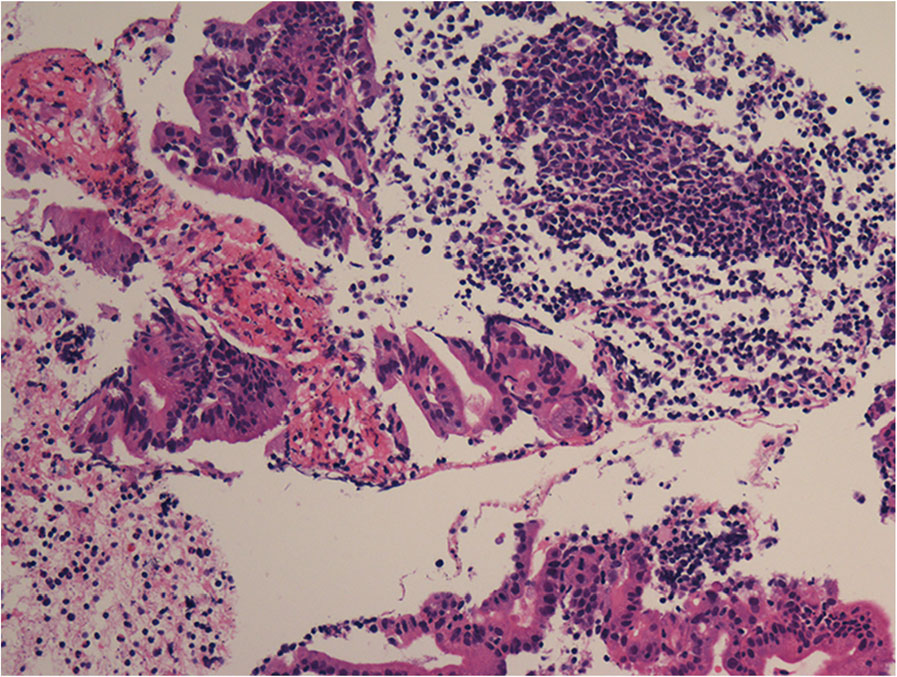
Histopathological findings of suspicious adenocarcinoma cells, H&E stain, magnification × 200

An endoscopic ultrasonography-guided puncture was performed, and biopsy found suspicious adenocarcinoma cells unexpectedly (Fig. 3). At the same time, a random biopsy of the two bumps with mucosa erosion near the pylorus on the anterior gastric antrum and pyloric mucosa revealed high-grade intraepithelial neoplasia, while gastroscopy showed normal appearance of the other gastric mucosae (Fig. 2b).

Treatment started with exploratory laparotomy. The whole stomach appeared soft, and no visible tumor was found. Local excision of the gastric wall was performed around the previous biopsy location, which was labeled by clips through a preoperative gastroscopy. The abdominal nodule was resected en bloc and sent for frozen section examination. Histological examination pointed out the consistent diagnosis of adenocarcinoma for both tissues. Then, a standard open distal radical gastrectomy (3 cm from the distal edge and 10 cm from the proximal edge of the lesion, including the gastric antrum mostly) and D2 lymph node dissection were performed. After the resection, the rest of the stomach was anastomosed with the duodenum.

**Fig. 4:**
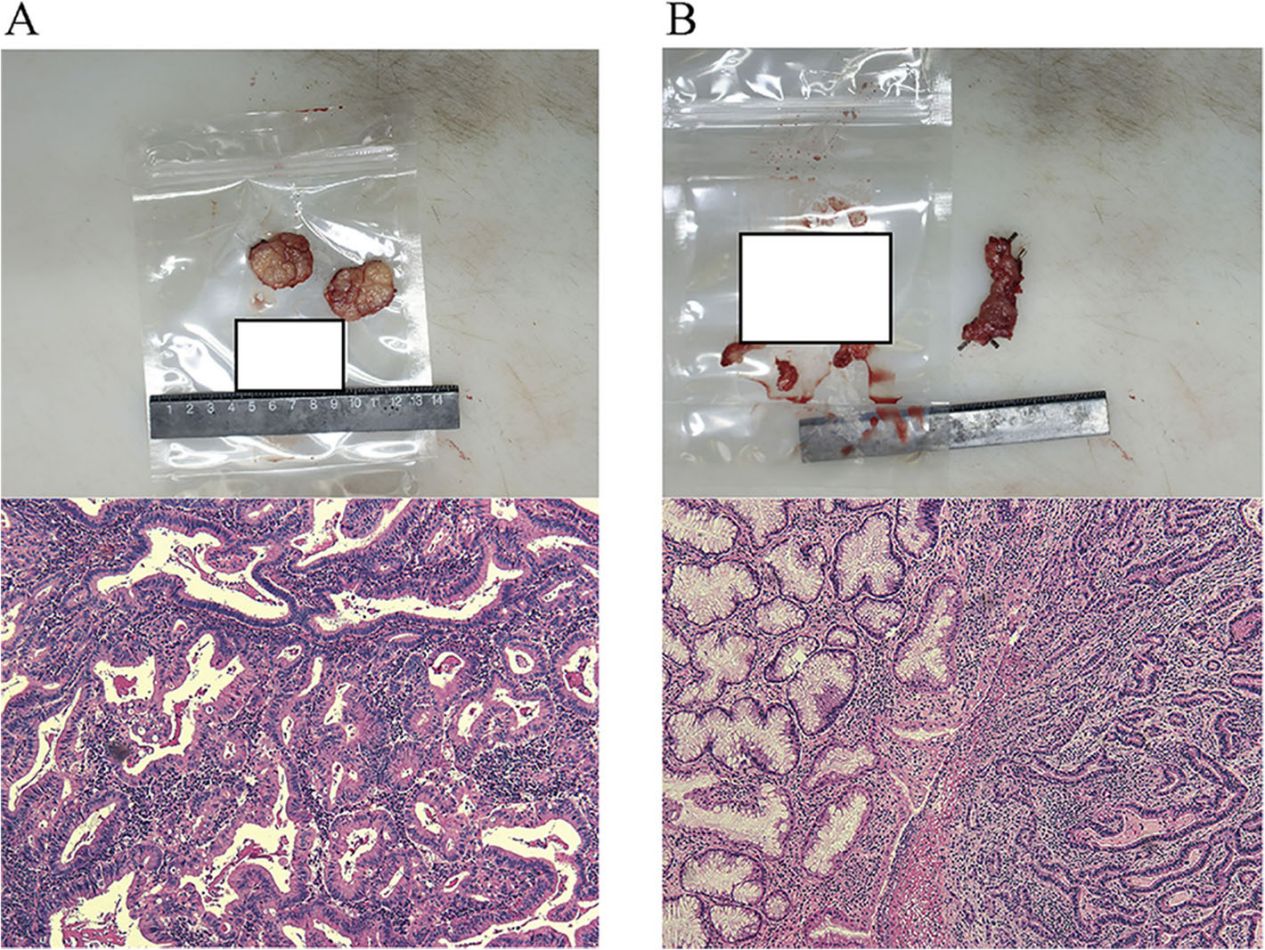
Gloss pictures and histopathological mappings (H&E stain, magnification × 100) of **a** the metastasized lymph node and **b** GC lesion

**Fig. 5:**
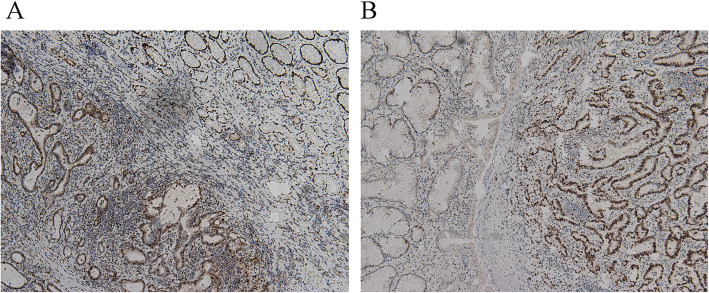
Immunohistochemical test showed positive MSG2 (**a**) and MSH6 (**b**)

The post-operation pathological examination of the lesions of the gastric wall (diameter, 0.3 cm) and the solitary nodule (3.5 cm) confirmed the diagnosis of highly to intermediately differentiated gastric adenocarcinoma (Fig. 4). Tumor tissue invasion was found in the deep muscularis without intravascular carcinoma thrombus and bundle invasion. Immunohistochemical test showed positive mutS homolog (MSH) 2 and MSH6 (Fig. 5) and negative human epidermal growth factor receptor (HER) 2, mutL homolog (MLH) 1, and mismatch repair system component PMS2. Interestingly, the previous “solitary nodule” was, in fact, merging five metastatic lymph nodes of the 8th group, while over 30 dissected lymph nodes of the other 11 groups were not involved. The final diagnosis was gastric antrum adenocarcinoma with lymphatic metastasis (T2N2M0, according to the 7th edition of the American Joint Committee on Cancer staging manual [4]).

The patient recovered soon and then took Tegafur capsules for therapy. Regular follow-up revealed her status as disease-free with good performance 14 months after the surgery.

## Discussion and conclusions

Lymph node metastasis has been proven as one of the most significant factors associated with unfavorable prognosis of GC patients [4]. The percentage of skip metastasis with solitary positive node ranges from 9.2 to 16.7%, in which the occult metastasis or micrometastasis could promote skip metastasis [5, 6]. Although the T stage is one of the independent risk factors for solitary lymph node metastasis (SLM) and an independent prognostic factor for survival, SLM is associated with the depth of tumor invasion and has prognostic significance for GC [7]. An asymptomatic, slow-growing solitary abdominal nodule often raises suspicion for benign lesions, such as solitary fibroma, Castleman’s disease, or stromal tumor. Therefore, it increased the difficulty to indicate the primary lesion when GC is already easy to be overlooked at times, even by expert gastroscopists [8]. The huge intra-abdominal solitary nodule, which was confirmed as a mass of merging skip metastatic lymph nodes, stayed stable for over 3 months without any therapy and observation of the primary GC, and this case in our experience is therefore of meaning.

The most frequent sites for skip metastases were the 7th, 8th, and the 9th lymph node stations [9]. The pathological examination of the present case confirmed SK of the 8th lymph node station, while over 30 dissected lymph nodes of the other 11 groups were not involved. The result supports the hypothesis of Choi et al. [3] that the less developed LNs cause the bypass or direct lymphatic flow to another involvement area in the skip groups.

Occult GC is an infrequent but not rare type of advanced GC, but the absence of clinical symptoms and physical sign related to the stomach always leads to a higher complexity for diagnosis. Meanwhile, a tiny minority of GC patients can be diagnosed as clinical occult GC with metastatic foci as the first finding, like pulmonary tumor embolism and bilateral adrenal enlargement [10–12], resulting in ignorance of the primary lesion. Literature reports a good performance of CECT in GC evaluation, with diagnostic accuracy that varies from 77 to 89% [13]. However, after the fault of several CECTs, the hinge point was the random biopsy of the pyloric mucosa that indicated high-grade intraepithelial neoplasia. Despite the occasionality, random gastric biopsy was helpful in finding the primary cancer in other cases, especially for the invisible GC [8, 14]. Some studies reported the utility of positron emission tomography/computed tomography with fluorodeoxyglucose (FDG PET/CT) to detect occult cancer and showed the higher efficacy than conventional imaging modalities [15, 16]. Meanwhile, some biomarkers, like HER2 [17], may play a potential role in detecting the occult GC and differential diagnosis as well. To our knowledge, this is the first case about occult GC with a big solitary skip metastatic lymph node. This case highlights the necessity of close observation and aggressive biopsy for intra-abdominal solitary nodules, even radiological suspicion of benign lesions. And this case reiterates the concentration on vigilance to the SK of GC and occult GC.

## Data Availability

The datasets used during the current study are available from the corresponding author on reasonable request.

## Abbreviations

CA: Carbohydrate antigen
CEA: Carcinoembryonic antigen
CECT: Contrast-enhanced abdominal computed tomography
FDG PET/CT: Positron emission tomography/computed tomography with fluorodeoxyglucose
GC: Gastric cancer
HER: Human epidermal growth factor receptor
LNM: Lymph node metastasis
MLH: MutL homolog
MSH: MutS homolog
PMS2: Mismatch repair system component
SLM: Solitary lymph node metastasis
SK: Skip lymphatic metastasis
